# Incomplete Lineage Sorting Is Common in Extant Gibbon Genera

**DOI:** 10.1371/journal.pone.0053682

**Published:** 2013-01-14

**Authors:** Jeffrey D. Wall, Sung K. Kim, Francesca Luca, Lucia Carbone, Alan R. Mootnick, Pieter J. de Jong, Anna Di Rienzo

**Affiliations:** 1 Institute for Human Genetics, University of California San Francisco, San Francisco, California, United States of America; 2 Department of Epidemiology and Biostatistics, University of California San Francisco, San Francisco, California, United States of America; 3 Department of Human Genetics, University of Chicago, Chicago, Illinois, United States of America; 4 Department of Behavioral Neuroscience, Oregon Health and Science University, Portland, Oregon, United States of America; 5 Gibbon Conservation Center, Santa Clarita, California, United States of America; 6 Children’s Hospital of Oakland Research Institute, Oakland, California, United States of America; North Carolina State University, United States of America

## Abstract

We sequenced reduced representation libraries by means of Illumina technology to generate over 1.5 Mb of orthologous sequence from a representative of each of the four extant gibbon genera (*Nomascus, Hylobates, Symphalangus, and Hoolock*). We used these data to assess the evolutionary relationships between the genera by evaluating the likelihoods of all possible bifurcating trees involving the four taxa. Our analyses provide weak support for a tree with *Nomascus* and *Hylobates* as sister taxa and with *Hoolock* and *Symphalangus* as sister taxa, though bootstrap resampling suggests that other phylogenetic scenarios are also possible. This uncertainty is due to short internal branch lengths and extensive incomplete lineage sorting across taxa. The true phylogenetic relationships among gibbon genera will likely require a more extensive whole-genome sequence analysis.

## Introduction

Gibbons (family Hylobatidae) are small, arboreal apes found in South, Southeast, and East Asia. They are the sister group to humans and the great apes, and share a common ancestor with other hominoids roughly 16–20 Mya [1]–[2]. Gibbons are divided into four well-recognized genera (*Hylobates*, *Nomascus*, *Symphalangus* and *Hoolock*) that have very different karyotypes and diploid chromosome counts which vary from 38 to 52.

**Figure 1:**
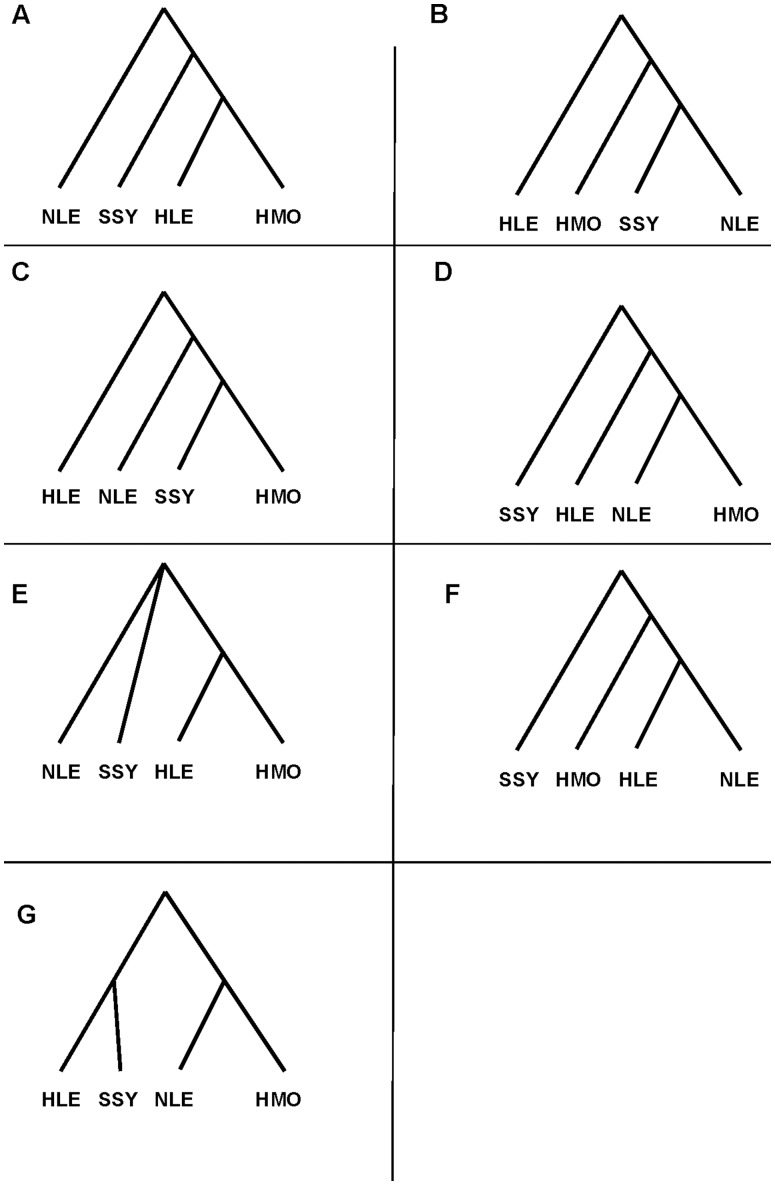
Schematic of different phylogenetic trees for the four gibbon genera. The trees represented in box A to F have been proposed as the results of previous studies and can be compared with the maximum-likelihood tree found in this study (G). (A) cf. [2], [7], [8], (B) cf. [6], [11], [12], (C) cf. [9], [10], (D) cf. [3], (E) cf. [4], [5], (F) cf. [28].

Despite a plethora of studies utilizing a range of different traits (e.g., vocalization, morphology, karyotype, mitochondrial DNA (mtDNA) variation, nuclear sequence variation), the phylogenetic relationships between gibbon species and genera remain unresolved (see Figure 1). Studies based on morphological and vocal traits have tended to support either *Nomascus* and *Hylobates* [3]–[4] or *Hoolock* and *Hylobates* [4]–[5] as sister taxa, while a study based on chromosomal rearrangements placed *Nomascus* and *Symphalangus* as sister taxa [6]. Most mtDNA studies have supported *Hylobates* and *Hoolock* as sister groups [2], [7], [8] but Takacs and colleagues [9] found support for a *Symphalangus* and *Hylobates* pairing. Studies of nuclear sequence variation (with or without mtDNA variation) have also been inconsistent, with *Symphalangus* and *Hylobates* [10] and *Symphalangus* and *Nomascus* [11]–[12] identified as possible sister taxa. Together, these studies have also differed in their identification of the most basal clade, with *Symphalangus*, *Hoolock* and *Nomascus* all proposed as outgroups to the other genera [3], [6], [7].

Part of the difficulty in assessing the true phylogenetic relationships between the four gibbon genera is that the initial time of divergence between the groups happened over a very short period of time. Due to incomplete lineage sorting, different genealogical trees will be ‘correct’ over different parts of the genome (i.e., gene trees are not necessarily concordant with species trees). So, for example, while it is now well established that our closest living relatives are chimpanzees and bonobos [13]–[15], there is still a small fraction of the genome where gorillas [14]–[15] or even orangutans [16] are our closest living relatives. In general, the true species tree and estimates of demographic parameters can only be recovered by jointly analyzing genetic data across many evolutionarily independent regions of the genome. While next-generation sequencing has greatly reduced the cost of gathering DNA sequence data in recent years, it is not so easy to harness this technology to generate orthologous data from taxa without a published reference genome sequence. (The draft gibbon genome sequence, while publicly available, is embargoed under the Ft. Lauderdale meeting agreement.) So, while sequencing whole genomes is now commonplace in humans [17] and in species with well-developed genomic resources [18]–[20], the largest extant study of sequence variation in the four gibbon genera looked at only 60 Kb of orthologous sequence data [12].

In this paper, we address the question of gibbon phylogeny by sequencing reduced representation libraries [21] to generate over 1.6 Mb of aligned sequence data from a single representative of each gibbon genus (Table 1). In brief, we digest genomic DNA using a restriction enzyme, size select the fragments, then sequence these fragments using next-generation sequencing (Illumina GAII). We then clustered sequences from different individuals to identify orthologs for downstream analyses. The same general approach can be used to analyze sequence data from non-model organism species without published genome sequences.

**Table 1:** Description of the individuals used in this study.

Scientific name	Individual	ISIS #	Gender	Abbreviation
Nomascus leucogenys	Asia	NL605	Female	NLE
Symphalangus syndactylus	Monty	SS910	Male	SSY
Hylobates moloch	Lionel	HMO894	Male	HMO
Hoolock leuconedys	Drew	HL307	Female	HLE

## Results and Discussion

### The Divergence between Gibbon Genera is Comparable with Levels of Divergence between Humans and Chimpanzees

Our clustering analyses produced a total of 30,484 reads that mapped uniquely to the single-copy portion of the human genome, with at least 20X coverage from each sample. We focus the remainder of our analyses to the 28,969 reads (1.59 Mb total length) that map to the human autosomes, since diploidy allows us to calculate both measures of genetic variation and genetic differentiation from our data.

Table 2 displays the average frequency of pairwise differences (π, cf. [22]) both within (π_w_) and between (π_b_) individuals. Both π_w_ and π_b_ were generally smaller than those reported in an earlier study using the same samples [12]. Since the current study is several times larger than the largest previous one, it is possible that the π values presented here are more accurate estimates of the true values. Alternatively, differences in the local sequence context of this study compared with our previous one [12] might explain the differences in observed levels of genetic diversity and genetic differentiation. However, there are two additional reasons why our methods might lead to systematic underestimates of π_w_ and π_b_: Orthologous regions with the highest levels of diversity/divergence may not be placed in the same cluster, and polymorphisms in the *RsaI* restriction site can cause allelic dropout, leading to loss of heterozygosity. Estimates from comparable human data suggest that allelic dropout leads to a ∼3% reduction in estimates of π [21].

**Table 2:** Average frequency of pairwise differences (%) within (π_w_) and between (π_b_) gibbon samples.

	HLE	NLE	SSY	HMO
**HLE**	0.106	1.198	1.176	1.197
**NLE**		0.204	1.228	1.236
**SSY**			0.151	1.225
**HMO**				0.174

If we take the π values at face value, then divergence between gibbon genera is on par with the levels of divergence between humans and chimpanzees [23], with sequence divergence times of 6–7 Mya, comparable to what was estimated from mtDNA [2]. Within-species diversity levels are similar to what has been reported for great apes [24], and slightly higher than what has been found in humans [17].

### A New Phylogenetic Tree for the Four Gibbon Genera

**Figure 2:**
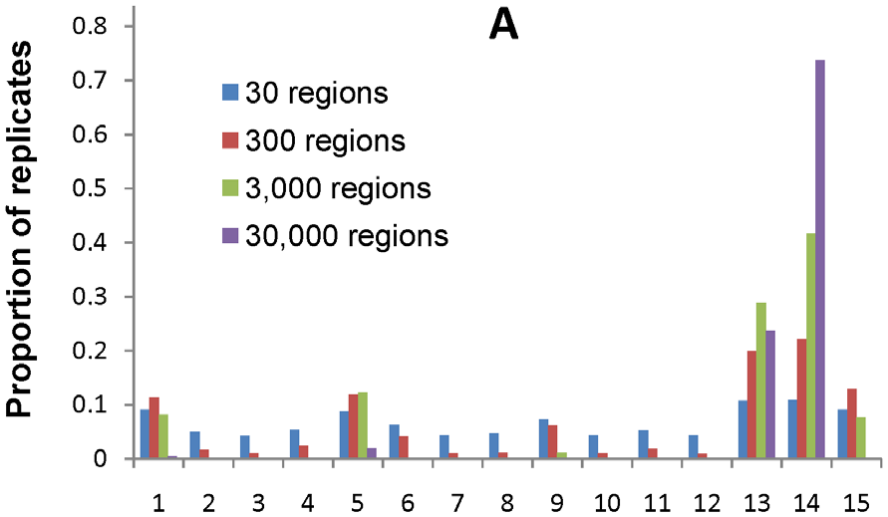
Distribution of the most likely bifurcating tree. (A) Distributions based on 10^5^ bootstrap replicates for 30, 300, 3,000 and 30,000 autosomal regions. (B) Key describing the bifurcating tree for the x-axis of Figure 2A.

Next, we used our data to examine the phylogenetic relationships between the four gibbon genera. Assuming evolutionary independence across regions, we used PAML [25] to calculate the most likely bifurcating topology. The maximum-likelihood tree had *Hoolock* and *Symphalangus* as sister taxa, and *Hylobates* and *Nomascus* as sister taxa (see Figure 1G). We obtained the same maximum-likelihood tree when we concatenated all of the regions together (i.e., no recombination between regions). This tree is different from all the previous trees proposed in the literature. To assess how much confidence we should place in our and other phylogenetic estimates, we bootstrap resampled from 30 to 30,000 regions from our data and tabulated the maximum-likelihood tree (using PAML) for each replicate (Figure 2). Our results suggest that sampling small amounts of data leads to substantial uncertainty in the final estimate. For example, when 300 regions are re-sampled (e.g., approximately 16 Kb of autosomal sequence data), 6 out of the 15 possible tree topologies are the maximum-likelihood tree in over 6% of the replicates. When we re-sample 3,000 or 30,000 regions, we recover the tree in Figure 1G in 42% and 74% of our replicates respectively (Figure 2). Most of the remaining replicates support the second most likely topology, with *Hoolock* and *Nomascus* as sister taxa, and *Symphalangus* and *Hylobates* as sister taxa. The tree shown in Figure 1G is also supported by a qualitative analysis of parsimony-informative sites (i.e., sites where exactly 2 out of 4 sampled sequences share a derived allele compared with the human reference genome hg19). There are significantly more sites that support *Hoolock* and *Symphalangus* as sister taxa (958 sites) than sites that support the other two unrooted trees (882 and 872 sites; *p*<0.05).

Discordance between gene trees at different genomic locations can arise due to two main causes: incomplete lineage sorting or recurrent mutations at the same nucleotide site (i.e., identity by state mistaken for identity by descent). We performed two additional analyses to assess the relative importance of these two potential explanations. First, we removed all CpG sites (known to have mutation rates ∼10 times higher than the genome-wide average) and repeated our analyses. Again, we found the tree shown in Figure 1G to be the most likely phylogeny. Also, we assessed how often parsimony-informative sites which supported the same phylogeny were found within the same fragment. If these sites were primarily due to recurrent mutation, then we would not expect any enrichment of multiple parsimony informative sites supporting the same phylogeny relative to a null model of random distribution of parsimony-informative sites. We observe a 4-fold enrichment of such ‘clustered’ informative sites, which is highly significant (*p*<<10^−6^).

We conclude that incomplete lineage sorting is the primary explanation for the observed patterns of genetic variation. So, while the maximum-likelihood tree obtained in this study must be considered provisional, we should have even less confidence in the phylogenetic trees estimated from previous studies based on smaller genetic data sets. This is particularly true for mtDNA, which due to a lack of intragenic recombination acts as a single genetic locus, and is a particularly poor region for phylogenetic studies in cases where short internal branches cause widespread incomplete lineage sorting.

### Conclusions

This study highlights the ability of next-generation sequencing technologies to be used to study patterns of within and between species genetic variation in non-model organisms without a published genome sequence. The protocol used is simple and inexpensive. It belongs to a set of methods using restriction enzyme digestion of target genomes to reduce the complexity of the target [26]. Given the recent decrease in sequencing costs over the past few years, it is now feasible for independent labs to conduct large-scale population genetic and molecular evolutionary studies in taxa without a reference genome sequence [27].

As proof-of-principle, we gathered over 1.5 Mb of orthologous sequence from representatives of all four gibbon genera. We used the data to explore the evolutionary relationships between our samples, and discovered that widespread incomplete lineage sorting makes it difficult to assess the true species tree from the amount of data that we gathered. While precise phylogenetic relationships will eventually be determined from even larger studies (e.g., the ongoing gibbon genome project), we caution that previous studies of gibbon taxonomy based on far less data than this study are likely to be inaccurate.

## Materials and Methods

Genomic DNA was isolated from blood from a single representative of each gibbon genus (Table 1). All samples were collected during routine health monitoring by Alan Mootnick, former director of the Gibbon Conservation Center (GCC), and the blood sampling was in keeping with the protocols approved by the GCC’s Animal Care and Use Committee. The GCC’s gibbons are kept in custom outdoor enclosures in Santa Clarita, CA. Each family group is kept in a separate enclosure, and each enclosure has branches and ropes to allow brachiation through the entire space. The gibbons are normally fed 10 times a day with up to 20 different types of food (primarily fruits and vegetables). Specific diets vary by individual need, preference and seasonal availability. The foods are alternated and presented in diverse ways to stimulate natural foraging behaviors and enrich the gibbons’ mental state.

We then constructed reduced representation libraries for each sample following the protocol of Luca and colleagues [21]. The DNA samples were digested by the restriction enzyme *RsaI* overnight, followed by size selection of fragments in the 70–80 bp range. The targeted DNA fragments were then isolated and purified from gel, and Illumina sequencing libraries were prepared using standard protocols. See [21] for further details. 72 base pair single-end reads were generated on an Illumina GAII, using 4, 3, 2 and 2 flow cell lanes for HLE, NLE, SSY and HMO respectively (see Table 1 for abbreviations). We obtained from 12–52 million sequence clusters from the four samples, with the smallest number coming from HLE.

Given the absence of a published gibbon reference sequence, we implemented a clustering method to categorize all sequence reads across the four samples into independent and discrete bins. We started by excluding reads that did not have at least 35 consecutive bases with PHRED quality scores >20. Then, we took the sample with the least amount of data, HLE, and aligned all HLE reads to each other. A read was put into a cluster if at least one pairwise alignment with a cluster member contained at most three mismatches. Using this criterion, we obtained 476,135 clusters for the HLE sequences. We then queried NLE, SSY and HMO reads against each of the HLE sequence bins and clustered them into the first identified bin with at most five mismatches. Possible overlap with multiple clusters is dealt with later (see below).

Using a mirrored version of the UCSC genome browser, we used BLAT to identify all BHO sequence bins that uniquely aligned to the human reference genome (build 19, commands stepSize = 5, repMatch = 2253). We then removed clusters that fit the following criteria:

- Clusters with <20X coverage
- Clusters that did not map to the human reference sequence
- Multiple clusters that mapped to the same location in the human reference sequence (if the clusters could not be combined)
- Clusters with <60 bp of alignment to the human reference sequence
- Clusters that aligned to >90 bp of the human reference sequence
- Clusters with a second-best alignment that had at least half the sequence match of the best alignment

A total of 30,484 clusters were left, comprising 2,175,349 total bases. For each gibbon, heterozygous alleles were called when a second allele was found in at least 20% of the sequence reads. Positions where third (plus fourth) alleles were found in 20% of the reads were converted to missing data.

To explore potential platform-specific sequencing biases, we plotted the frequency of SNPs as a function of the base position on each read. We find an excess of (apparent) polymorphisms that are located near the beginning and towards the ends of the sequence reads, as well as an increase in uncalled bases towards the ends of the reads (Results not shown). These results are consistent with the known biases associated with restriction enzyme digested reads, as well as the decrease in sequence quality near the end of next-generation sequencing reads. To reduce the effect of these biases on our data analyses, we trim the first 2 and the last 15 bases from each read, leaving 30,484 reads covering a total of 1,668,456 bases.

For each read cluster, we mapped the reads onto the human reference genome (hg19). We then used PAML version 4 [25] to estimate the likelihood of each of the 15 different bifurcating trees using default parameters (and with the hg19 allele as the outgroup). To calculate the likelihoods for each tree using the whole autosomal data set, we multiply the likelihoods for each autosomal read together (i.e., we assume evolutionary independence across regions). Finally, for the bootstrap resampling, we resampled 30–30,000 regions (with replacement) 10^5^ times, multiplying the likelihoods across sampled regions.
